# Diversity and function of multicopper oxidase genes in the stinkbug *Plautia stali*

**DOI:** 10.1038/s41598-020-60340-8

**Published:** 2020-02-26

**Authors:** Yudai Nishide, Daisuke Kageyama, Masatsugu Hatakeyama, Kakeru Yokoi, Akiya Jouraku, Hiromitsu Tanaka, Ryuichi Koga, Ryo Futahashi, Takema Fukatsu

**Affiliations:** 1 National Agriculture and Food Research Organization (NARO), Institute of Agrobiological Sciences Ohwashi, Tsukuba, 305-8634 Japan; 2 National Institute of Advanced Industrial Science and Technology (AIST), Tsukuba, 305-8566 Japan; 3 Department of Biological Sciences, Graduate School of Science, University of Tokyo, Tokyo, 113-0033 Japan; 4 Graduate School of Life and Environmental Sciences, University of Tsukuba, Tsukuba, 305-8572 Japan

**Keywords:** Entomology, Evolutionary genetics

## Abstract

Multicopper oxidase (MCO) genes comprise multigene families in bacteria, fungi, plants and animals. Two families of MCO genes, MCO1 (laccase1) and MCO2 (laccase2), are conserved among diverse insects and relatively well-characterized, whereas additional MCO genes, whose biological functions have been poorly understood, are also found in some insects. Previous studies reported that MCO1 participates in gut immunity and MCO2 plays important roles in cuticle sclerotization and pigmentation of insects. In mosquitoes, MCO2 was reported to be involved in eggshell sclerotization and pigmentation, on the ground that knockdown of MCO2 caused deformity and fragility of the eggshell. Here we identified a total of 7 MCO genes, including *PsMCO1* and *PsMCO2*, and investigated their expression and function in the brown-winged green stinkbug *Plautia stali*. RNA interference (RNAi) knockdown of MCO genes by injecting double-stranded RNA (dsRNA) into nymphs revealed that MCO2, but not the other 6 MCOs, is required for cuticle sclerotization and pigmentation, and also for survival of *P. stali*. Trans-generational knockdown of MCO2 by injecting dsRNA into adult females (maternal RNAi) resulted in the production of unhatched eggs despite the absence of deformity or fragility of the eggshell. These results suggested that MCO2 plays an important role in sclerotization and pigmentation of the cuticle but not in eggshell integrity in *P. stali*. Maternal RNAi of any of the other 6 MCO genes and 3 tyrosinase genes affected neither survival nor eggshell integrity of *P. stali*. Contrary to the observations in the red flour beetle and the brown rice planthopper, RNAi knockdown of MCO6 (MCORP; Multicopper oxidase related protein) exhibited no lethal effects on *P. stali*. Taken together, our findings provide insight into the functional diversity and commonality of MCOs across hemipteran and other insect groups.

## Introduction

Enzymes of the multicopper oxidase (MCO) family comprise a group of proteins that are ubiquitously found in diverse organisms. Biological functions of MCOs have been characterized in bacteria, fungi, plants and animals including insects^1^. MCOs have a broad substrate range and perform a wide variety of tasks such as pigmentation, lignin synthesis and degradation, iron homeostasis, and morphogenesis. Laccases, representing the largest subgroup of MCOs, are enzymes with p-diphenol oxidase activity. In fungi, laccases have been studied extensively because they have a broad range of biotechnological applications including wood delignification for paper manufacturing, dye destaining in textile industry, and detoxification of water pollutants^2,3^.

Insect cuticle, an extracellular material consisting of a network of chitin fibers embedded in a protein matrix, is a main component of the integument, which serves to protect the insects against pathogens and physical impacts. In the insect cuticle, two types of enzymes, tyrosinases and MCOs (collectively called phenoloxidase), are involved in melanin synthesis^4^. For example, the red flour beetle *Tribolium castaneum* (Coleoptera: Tenebrionidae) has 3 MCO-like genes (MCO1 formerly called laccase1, MCO2 also called laccase2, and MCO-related protein (MCORP)) and 2 tyrosinase-like genes. Among them, MCO2 (laccase2) is considered to have a conserved function in cuticle pigmentation and sclerotization across diverse insects, on the ground that RNAi knockdown of MCO2 (laccase2) causes soft and unpigmented cuticle and physical deformities in *T. castaneum*^5,6^, the longhorn beetle *Monochamus alternatus* (Coleoptera: Cerambycidae)^7^, the western corn rootworm *Diabrotica virgifera virgifera* (Coleoptera: Chrysomelidae)^8^, the fruit fly *Drosophila melanogaster* (Diptera: Drosophilidae)^9^, the honeybee *Apis mellifera* (Hymenoptera: Apidae)^10^, the two-spotted cricket *Gryllus bimaculatus* (Orthoptera: Gryllidae)^11^, the stinkbugs *Riptortus pedestris*, *Megacopta punctatissima*, *Nysius plebeius* and *Oncopeltus fasciatus* (Hemiptera: Alydidae, Plataspidae and Lygaeidae)^12,13^, the termites *Reticulitermes speratus* and *Zootermopsis nevadensis* (Isoptera: Rhinotermitidae)^14,15^ and the dragonfly *Nannophya pygmaea* (Odonata: Libellulidae)^16^.

MCO2 exhibits high levels of expression in eggs and ovaries of mosquitos *Culex pipiens*^17^ and *Anopheles gambiae* (Diptera: Culicidae)^18,19^, suggesting the possibility that MCO2 may play a role in eggshell integrity. This idea was confirmed in a different mosquito, *Aedes albopictus*, in which RNAi knockdown of MCO2 inhibited eggshell pigmentation and sclerotization, resulting in the production of whitish and deformed eggs^20^. It still remains to be explored whether MCO2 is also involved in eggshell pigmentation and sclerotization in other insect groups.

MCO1 is another multicopper oxidase found in all the insect genomes sequenced to date^21^. In *A. gambiae*, MCO1 is constantly expressed throughout the developmental course and the expression is pronounced in the midgut and Malpighian tubules at the adult stage^18^. Up-regulation of MCO1 in response to immune challenge or blood meal suggested that MCO1 may be involved in endogenous iron homeostasis and/or gut immunity^18^. Involvement in endogenous iron homeostasis has been suggested in *D. melanogaster*^22,23^ and involvement in gut and humoral immunity has been also suggested in the whitefly *Bemisia tabaci* (Hemiptera: Aleyrodidae)^24^, the grain aphid *Sitobion avenae* (Hemiptera: Aphididae)^25^ and the diamondback moth *Plutella xylostella* (Lepidoptera: Plutellidae)^26^.

In addition to MCO1 and MCO2, other MCO genes have been identified in some insects. In the brown rice planthopper *Nilaparvata lugens* (Hemiptera: Delphacidae), for instance, 7 MCO genes (MCO1–MCO7) were identified by genomic and transcriptomic analyses^27^. Some specialized roles in reproduction was suspected for MCO3 in *N. lugens*, on the ground that MCO3 is specifically and highly expressed in ovaries only at the adult stage. However, RNAi knockdown of MCO3 exhibited little effects on ovarial development, fecundity and egg hatchability of *N. lugens*. Intriguingly, Ye *et al*. (2015) showed that RNAi knockdown of MCO6 (MCORP) resulted in lethality of *N. lugens*, in which the expression pattern of MCO6 during the developmental course was similar to that of MCO2 (laccase2).

The brown-winged green stinkbug *Plautia stali* (Hemiptera: Pentatomidae) is known as a devastating pest of various fruits and crops^28^. Here, in an attempt to gain further insight into the diversity and function of MCOs, we surveyed and identified a total of 7 MCO genes, including MCO1 and MCO2, from *P. stali*, and investigated their expression and function during the developmental course.

## Materials and Methods

### Insects

Adult insects of *P. stali*, collected at a forest edge in the National Institute of Advanced Industrial Science and Technology, Tsukuba, Japan, were used to establish an inbred laboratory strain. A mass-reared colony of the strain was used as the source of the experimental insects. The insects were reared in plastic containers (150 mm in diameter, 60 mm in height) with raw peanuts, dry soybeans, and water supplemented with 0.05% ascorbic acid at 25 °C ± 1 °C under a long-day regime of 16 h light and 8 h dark as previously described^29^.

### Characterization of MCO genes in *P. stali*

Total RNAs were extracted from the whole body of adult females approximately 5 days after ecdysis using RNeasy Mini Kit (Qiagen, Hilden, Germany). The complimentary DNAs (cDNAs) were sequenced by Illumina HiSeq. 2500 with paired end 101 bp (Macrogen Japan Corp., Kyoto, Japan), and the generated raw reads (Accession nos. DRR118506–DRR118507) were analyzed as previously described^30^. The assembled sequences were subjected to BLASTx, by which 7 MCO genes (accession numbers LC495719–LC495725) and 3 tyrosinase-like genes (accession numbers LC495726–LC495728) were identified. On the basis of each predicted sequence, we designed primer pairs for quantitative RT-PCR and RNAi (Tables S1 and S2).

### Phylogenetic analyses

The deduced amino acid sequences of MCO genes of *P. stali* and other insects were aligned using the Clustal W program implemented in MEGA 5.2^31^. The molecular phylogenetic analyses were conducted by the maximum likelihood and neighbor-joining methods using MEGA 5.2 with Jones-Taylor-Thornton model (default setting). Bootstrap values based on 1,000 replications are shown as percentages on the nodes.

For comparison of multicopper-coordinating residues, we produced an alignment of MCO and MCORP genes using muscle program in MEGA 5.2. To identify copper coordinate residues, yeast multicopper ferroxidase, Fet3p, was used as a query. Fet3p contains 10 histidines and 1 cysteine that coordinate with four coppers^32^. T1 copper is coordinated with H413, C484 and H489, T2 copper with H81 and H416, T3α copper with H83, H126 and H485, and T3β copper with H128, H418 and H483.

### RNAi knockdown of MCO genes

Template preparation for double-stranded RNA (dsRNA) synthesis was performed by PCR using the primers designed (Table S2) in combination with T7 promoter sequence at the 5-prime end. The PCR products were purified using QIAquick gel extraction kit (QIAGEN, Hilden, Germany) and subjected to transcription into dsRNA using RiboMAX Large Scale RNA Production Systems (Promega, Wisconsin, USA).

To suppress the mRNA levels of MCO genes, newly-molted (within 1 day after molting) third or fifth (last) instar nymphs were injected with dsRNA solution (around 1 or 3 µl at a concentration of 100 ng/µl) into the ventral septum between the thoracic and abdominal segments.

To estimate mRNA suppression efficiency, total RNAs extracted from whole bodies of fifth instar nymphs 3 days after injection were reverse-transcribed into cDNA using High-Capacity cDNA Reverse Transcription Kit (Thermo Fisher Scientific, Massachusetts, USA). Quantitative reverse transcription (RT) PCR of the MCO genes was conducted using LightCycler 480 or LightCycler 96 with LightCycler 480 SYBR Green Master (Roche Diagnostics, Basel, Switzerland).

### Effects of maternal RNAi of MCOs

For maternal RNAi, dsRNA solution of *PsMCO2* (*Pslaccase2*) (5 µl at a concentration of 100 ng/µl) was injected into adult females approximately 2 weeks after ecdysis, which were reproductively mature and starting oviposition. The deposited egg masses were collected and individually placed in a 35 mm plastic Petri dish with a cotton ball soaked with water. Egg hatching rate was determined for each egg mass by counting the total number of eggs and the number of hatched nymphs.

Maternal RNAi of 6 MCOs other than MCO2 (laccase2) and 3 tyrosinase genes was also performed in the same method as maternal RNAi of MCO2 as described above. The deposited egg masses were collected daily and the number of egg masses and the number of eggs in each egg mass were counted. Egg hatching rates were determined by counting the total number of eggs and the number of hatched nymphs.

To estimate maternal RNAi efficiency, egg masses laid 4 or 5 days after injection were collected individually and then, 3 days later, the total RNA of each egg mass was extracted. Quantitative RT-PCR was conducted as described above.

### Fluorescent microscopy

The *PsMCO2* dsRNA was labelled with Cy3 fluorochrome using a Label IT Cy3 labelling kit (TaKaRa Bio Japan, Inc., Shiga, Japan). Adult females approximately 2 weeks after eclosion, which had already started oviposition, were injected with 5 µl of Cy3-labelled dsRNA. The females 3 days after injection were dissected in phosphate buffered saline (PBS; 137 mM NaCl, 2.7 mM KCl, 10 mM Na_2_HPO_4_, 1.8 mM KH_2_PO_4_ [pH 7.4]) and the abdominal part containing ovaries were photographed under a stereomicroscope (MZ16F; Leica, Wetzlar, Germany) equipped with fluorescent optics with a DsRed filter.

### Ovary and egg samples

Immature ovaries were collected from newly emerged adults, whereas mature ovaries and fully developed oocytes were collected from reproductively mature 14-day-old adults. Fully developed oocytes showing typical form of eggs were taken from mature ovaries and the remaining parts of the ovaries were used as “mature ovaries” samples. Egg samples were collected daily after oviposition (1d–5d).

## Results and Discussion

### Identification of 7 MCO genes in *P. stali*

**Figure 1:**
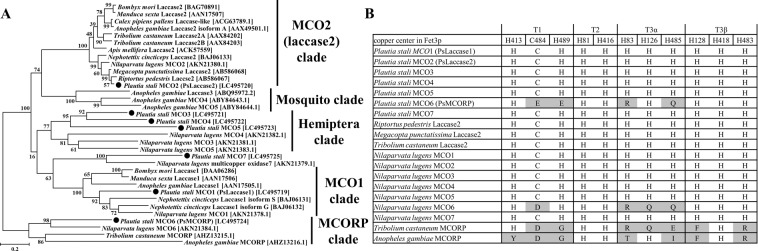
(**A**) Phylogenetic tree of MCO genes based on deduced amino acid sequences. The tree was inferred from 406 aligned amino acid sites using the maximum likelihood method. Bootstrap values based on 1,000 replications are shown as percentages on the nodes. Black ovals indicate the MCO genes of *P. stali* and accession numbers are shown in brackets. (**B**) Amino acid comparison of multicopper-coordinating residues of MCO and MCORP genes. Yeast multicopper ferroxidase, Fet3p, contains 10 histidines and 1 cysteine that coordinate with four coppers, T1, T2, T3α and T3β^32^. Some copper coordinate residues were missing in MCO6 and MCORPs. The cells showing non-conserved histidine or cysteine were highlighted in gray.

In order to identify and characterize MCO genes from the transcriptome of *P. stali*, BLASTx searches and molecular phylogenetic analyses were performed together with MCO orthologs reported from other insect species on the basis of their putative amino acid sequences. On the maximum likelihood tree, one MCO gene of *P. stali* fell into the well-supported clade of MCO1 (or laccase1), and another MCO gene was placed within the well-supported clade of MCO2 (or laccase2) (Fig. 1A). These genes were designated as *PsMCO1* (*Pslaccase1*) and *PsMCO2* (*Pslaccase2*), respectively. Among the other 5 MCO genes of *P. stali*, 3 genes (*PsMCO3*, *PsMCO4* and *PsMCO5*) formed a clade, which was adjacent to the clade consisting of MCO3, MCO4 and MCO5 of *N. lugens*, whereas 2 genes (*PsMCO6* and *PsMCO7*) were orthologous to MCO6 and MCO7 of *N. lugens*, respectively. *PsMCO6* (*PsMCORP*) and MCO6 of *N. lugens* were allied to the clade of multicopper oxidase related proteins (MCORP) (Fig. 1A). The topology of the neighbor jointing tree was substantially identical except for the relationship between MCO3, 4 and 5 of *N. lugens* (Fig. S1).

Almost all MCOs are known to contain 10 conserved histidine residues and one conserved cysteine residue that form copper centers^32^. The putative amino acid sequences of 6 MCOs of *P. stali* (*PsMCO1*, PsMCO2, *PsMCO3*, *PsMCO4*, *PsMCO5* and *PsMCO7*) retained these conserved residues (Fig. 1B). By contrast, in *PsMCO6* belonging to the MCORP clade, T2 and T3β copper coordinate residues were conserved but T1 and T3α copper coordinate residues were not (Fig. 1B). Notably, all members of the MCORP clade do not have T1 and T3α copper coordinate residues. In *T. castaneum*, it was reported that MCORP does not conserve T1, T3α and T3β copper coordinate residues and shows no oxidase activity^33^.

### Effects of RNAi knockdown of MCO genes in nymphal period

**Figure 2:**
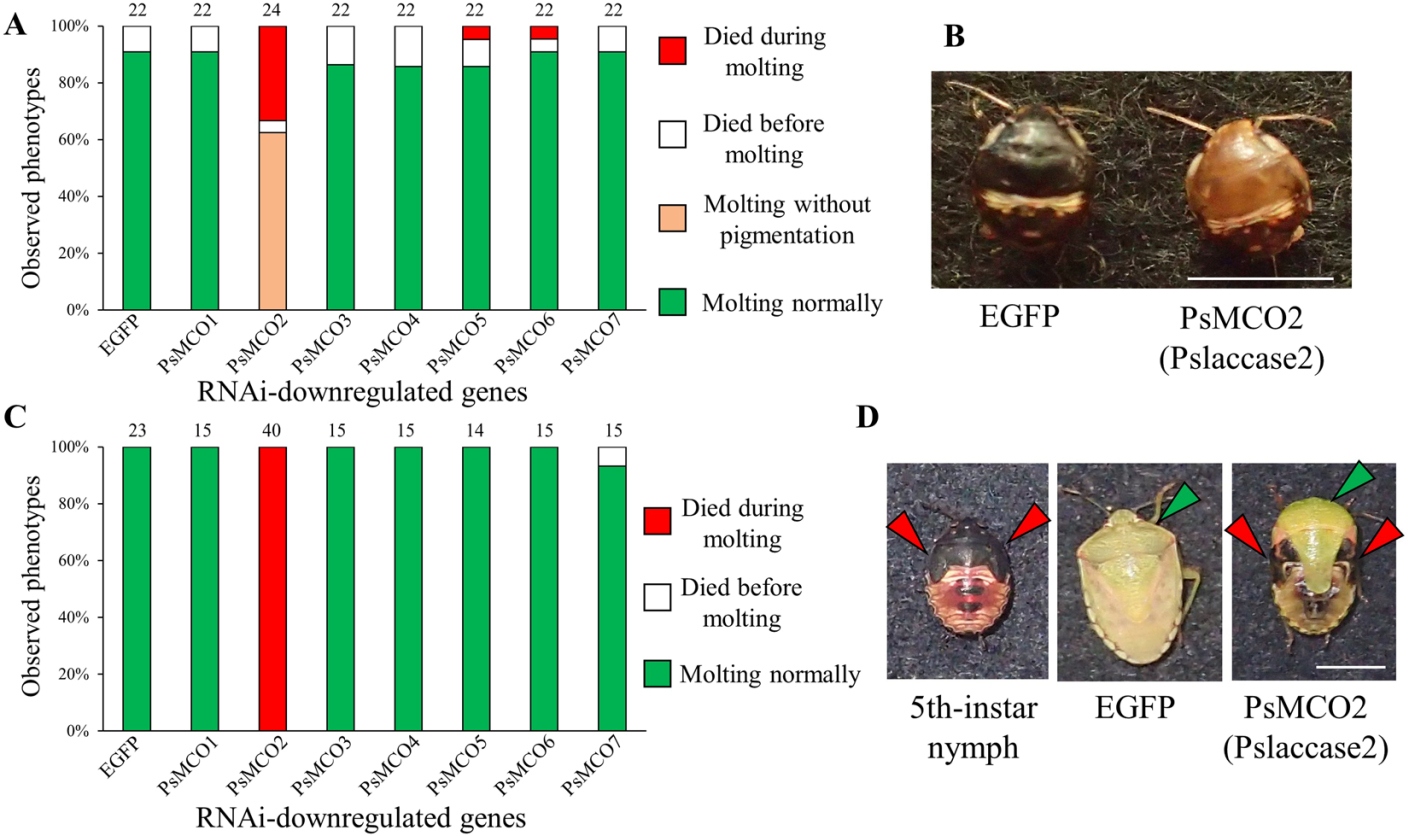
Effects of RNAi knockdown of MCO genes on nymphs of *P. stali*. (**A**) Effects of RNAi on third instar nymphs, in which dsRNA for each MCO gene was injected into newly molted third instar nymphs. Note that knockdown of *PsMCO2* led to high mortality during molting. (**B**) Phenotypes of newly molted fourth instar nymphs that were injected with either EGFP dsRNA as a control (left panel) or *PsMCO2* dsRNA (right panel). All the *PsMCO2* dsRNA-injected insects were not tanned. (**C**) Effects of RNAi on fifth instar nymphs. All the *PsMCO2* dsRNA-injected insects died during molting. (**D**) A fifth instar nymph (left), a 1-day-old adult that were injected with EGFP dsRNA as a control (middle), and a typical image of molting failure induced by injection with *PsMCO2* dsRNA (right). *PsMCO2* RNAi causes incomplete molting. Arrowheads indicate nymphal (red) and adult (green) cuticles. Numbers above bars show sample sizes (i.e. biological replicates) in. (**A**,**C**) Scale bars show 5 mm in (**B**,**D**).

To investigate the biological roles of the MCO genes, we injected dsRNA prepared for each of the MCO genes into newly-molted third or fifth instar nymphs of *P. stali*. Of 24 nymphs at the third instar stage that were injected with dsRNA of *PsMCO2*, 8 nymphs (33.3%) died during molting (Fig. 2A), whereas 15 nymphs (62.5%) reached fourth instar but remained soft and unpigmented, being unable to feed and eventually died within a week (Figs. 2B and S2). Normally, newly molted nymphs are soft and pinkish in color but promptly harden and darken within a few hours. The observed phenotype is similar to other cases of hemipteran insects, such as *R. pedestris*^12^ and *N. lugens*^27^, wherein RNAi of MCO2 was performed.

On the other hand, all the fifth instar nymphs injected with *PsMCO2* dsRNA (40 nymphs) died during molting without reaching adulthood (Figs. 2C,D and S3). Molting success among 3rd instar and 5th instar larvae is significantly different (Fisher’s exact test; p < 0.001). Although speculative, the severer phenotype in fifth instar nymphs compared with third instar nymphs is probably due to the fact that adult molting entails much more drastic change of body design than nymphal molting even in hemimetabolous insects. These results indicate that *PsMCO2* is necessary for cuticle tanning and hardening in *P. stali* as reported in many other insects^21^.

On the other hand, RNAi knockdown of other *PsMCO* genes did not affect normal molting and pigmentation in both third and fifth instar nymphs (Fig. 2), although quantitative RT-PCR confirmed that RNAi effectively worked for all the MCO genes (Fig. S4). Notably, although it was reported that RNAi knockdown of MCO6 in *N. lugens* and MCORP in *T. castaneum* causes high mortality^27,33^, RNAi knockdown of *PsMCO6* did not affect the survival of *P. stali* (Fig. 2).

### Effects of maternal RNAi of *PsMCO2*

**Figure 3:**
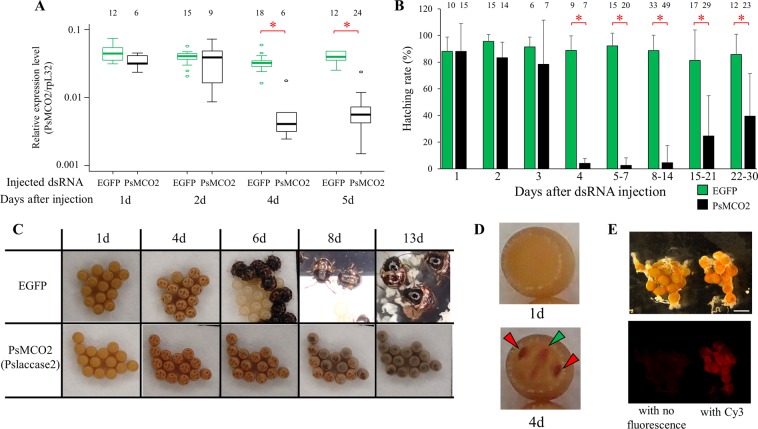
Effects of maternal RNAi knockdown of *PsMCO2* gene. (**A**) Expression levels of *PsMCO2* in embryos estimated by quantitative RT-PCR in terms of *PsMCO2* cDNA copies per ribosomal protein L32 (rpL32) cDNA copies. RNA was extracted from day 4 egg masses that were laid by females 1, 2, 4 and 5 days after dsRNA injection. (**B**) Egg hatching rates. Sexually mature females were injected with either EGFP dsRNA as a control or *PsMCO2* dsRNA and hatching rate of each egg mass was observed. Hatching rates significantly differ between the control eggs and the *PsMCO2* knockdown eggs from 4 days after injection and onward. Mean egg hatching rates of egg masses are shown with standard deviations. Numbers above bars show sample sizes (i.e. biological replicates). (**C**) Phenotypes of eggs laid by females injected with either EGFP dsRNA as a control or *PsMCO2* dsRNA. Eyespots and egg bursters are seen through the eggshell of 4-day-old eggs irrespective of the treatments. Subsequently, however, eggs laid by females injected with *PsMCO2* did not hatch (6d) and became blackened (8d). (**D**) Close-up view of an egg from a mother that was injected with *PsMCO2* dsRNA. The developed egg (fourth day after egg laying; bottom) shows obvious eyespots and an egg burster, whereas the undeveloped egg (first day after egg laying; upper) does not. Arrowheads indicate eyespots (red) and egg-burster (green). (**E**) Ovaries of sexually mature females injected with Cy3-labelled dsRNA (right) or non-labelled dsRNA (left), which are observed under light microscope (upper) and fluorescent microscope (bottom). Scale bar shows 1 mm.

When dsRNA of *PsMCO2* was injected into mated females of *P. stali*, the level of *PsMCO2* transcripts was significantly lower in eggs laid during 4 to 5 days after injection (t-test; *P* < 0.001; Fig. 3A), although the *PsMCO2* transcripts remained at the normal level in eggs laid during 1 to 2 days after injection. These results suggest the possibility that dsRNA injected into female adult hemocoel are incorporated into young oocytes but not into mature oocytes that are to be oviposited within 2 days.

Remarkably, RNAi knockdown of *PsMCO2* caused lethal effects on embryos, which was coincident with the downregulated *PsMCO2* transcription – eggs laid 1–2 days after injection hatched normally but eggs laid 4 days after injection and onward failed to hatch (t-test; *P* < 0.01; Fig. 3B). The effect of RNAi of *PsMCO2* on the egg hatching failure persisted for as long as 4 weeks, though attenuated 15 days after injection and onward. When eggs subjected to maternal RNAi of *PsMCO2* were compared with control eggs, no difference was recognized in appearance and elasticity of the eggs, which was roughly evaluated by pricking the eggshell with fine needles. These results suggest that *PsMCO2* is necessary for egg hatching but not for eggshell sclerotization. Notably, most unhatched embryos developed to a considerable extent within the eggshell, on the ground that their eyespots and egg bursters (hatching spines) were clearly seen through the eggshell (Fig. 3C,D). Plausibly, *PsMCO2* is essential for the embryonic development at a stage later than eyespot and egg-burster formation.

Previous studies suggested that MCO2 (laccase2) is necessary for formation and pigmentation of eggshell structure in mosquitoes^17,19,20^, which does not agree with our observations in *P. stali*. These results probably reflect the fact that the mechanisms underlying eggshell pigmentation and sclerotization in the hemipteran *P. stali* are not the same as those in the dipteran mosquitos.

To further confirm the efficacy of maternal RNAi, we attempted to visualize the uptake of dsRNA to ovaries. Cy3-labelled dsRNA of *PsMCO2* was injected into the body cavity of ovipositing adult females. Three days later, fluorescence was detected in the ovaries of the injected insects (Fig. 3E). This signal was considered to be derived from the Cy3-labelled dsRNA rather than autofluorescence, because the signal was almost undetectable in the ovaries of ovipositing females injected with non-labelled dsRNA (Fig. 3E) or fluorescence-labelling reagent alone (data not shown). These results indicate that the injected dsRNA can reach the ovaries and cause maternal RNAi therein. It is worth noting that the maternal organ with the most intense Cy3 signals was the accessary glands associated with the ovaries (Fig. S5), suggesting the possibility that the accessory glands may be involved in the uptake of dsRNA. The accessory glands produce the seminal fluid in males, which is reported to modulate the longevity and reproductive behavior of mated females in some insects^34^. Meanwhile, the function of the accessory glands is relatively ambiguous in females^35^. The high uptake of dsRNA is suggestive of some unknown function of the accessory glands.

### Effects of maternal RNAi of other MCO genes

**Figure 4:**
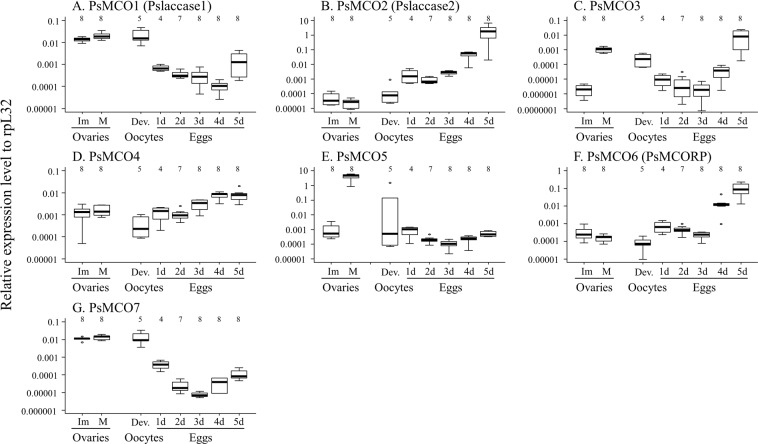
Expression patterns of 7 MCO genes in ovaries and eggs of *P. stali* in terms of cDNA copies per ribosomal protein L32 (rpL32) cDNA copy. (**A**) *PsMCO1* (*Pslaccase1*). (**B**) *PsMCO2* (*Pslaccase2*). (**C**) *PsMCO3*. (**D**) *PsMCO4*. (**E**) *PsMCO5*. (**F**) *PsMCO6* (*PsMCORP*). (**G**) *PsMCO7*. Immature ovaries (Im) were collected from newly emerged adults, whereas mature ovaries (M) and fully developed oocytes (Dev.) were collected from 14-day-old adults. Egg samples were collected daily after oviposition (1d–5d). Numbers above bars are sample size (i.e. biological replicates).

On account of the normal eggshell integrity upon maternal RNAi of *PsMCO2*, we examined the possibility that other MCOs might be involved in eggshell pigmentation and sclerotization in *P. stali*. In ovaries, expression levels of *PsMCO3* and *PsMCO5* increased drastically upon ovarian maturity (Fig. 4C,E), suggesting the possibility of some involvement in eggshell construction. Regardless of ovarian maturity, *PsMCO1* and *PsMCO7* exhibited relatively high expression levels (Fig. 4A,G), whereas *PsMCO2*, *PsMCO4* and *PsMCO6* maintained relatively low expression levels (Fig. 4B,D,F). In oocytes and eggs, the levels of *PsMCO1* and *PsMCO7* transcripts declined in the course of embryogenesis (Fig. 4A,G). By contrast, *PsMCO2* exhibited initially very low and subsequently increasing expression patterns in the course of embryogenesis (Fig. 4B), which seems concordant with the above observations that the *PsMCO2* knockdown resulted in the production of non-hatching eggs although embryonic development proceeded to a considerable extent within the eggs (Fig. 3). In the mosquito *A. gambiae*, expression levels of MCO2 are high in the eggs between 1 and 2 days old^18^. Because the embryonic period is over 3 days at general rearing temperature in *A. gambiae*^36^, the timing of MCO2 expression seems relatively earlier compared to *P. stali*. This temporal difference might explain the fact that maternal RNAi of MCO2 affects eggshell integrity in the mosquito^20^ but not in *P. stali*. *PsMCO6* showed similar expression patterns to *PsMCO2*, wherein high expression levels were found only in eggs at later developmental stages. Similar expression patterns of MCO2 and MCO6 were also observed in *N. lugens*^27^. *PsMCO3* showed bimodal expression patterns with peaks in embryos at early and late stages, while *PsMCO4* and *PsMCO5* exhibited relatively small changes regardless of the embryonic stages. Considering the relatively high expression levels in mature ovaries and/or fully developed oocytes, we regarded *PsMCO1*, *PsMCO3*, *PsMCO5* and *PsMCO7* as candidate genes that may contribute to the eggshell integrity.

**Figure 5:**
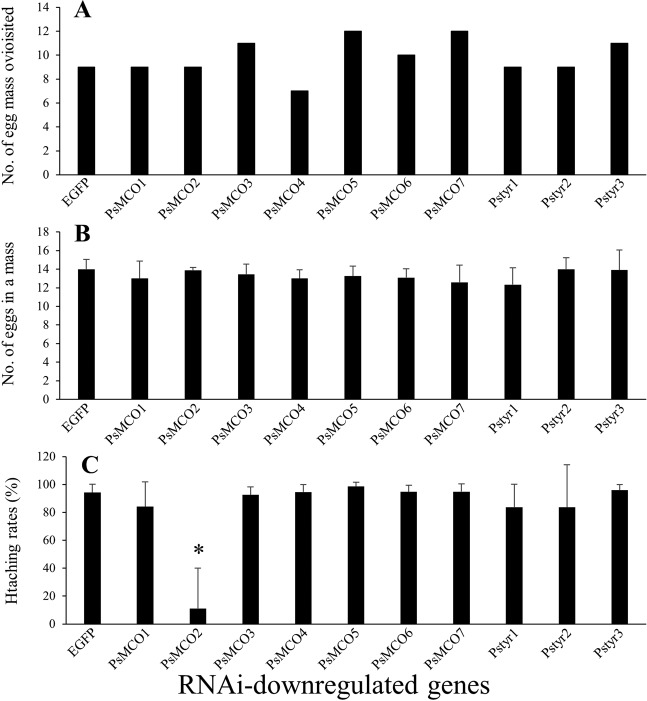
Effects of maternal RNAi knockdown of 7 MCO and 3 tyrosinase genes on egg production and hatching. Six sexually mature females were injected with each MCO or tyrosinase dsRNA. Total number of egg masses oviposited from 4 to 7 days after injection (**A**), mean number of eggs in each egg mass (**B**) and egg hatching rates (**C**) are shown. No significant differences were observed among them except for the hatching rate for RNAi of *PsMCO2* (Steel-Dwass test; *P* < 0.05).

However, maternal RNAi of any of *PsMCO1* to *PsMCO7*, as well as *PsTyr1* to *PsTyr3* (tyrosinase, i.e., another phenoloxidase), did not cause such phenotypes as whitish and deformed eggshell, although quantitative RT-PCR confirmed that maternal RNAi effectively worked in eggs for all the MCO genes (Fig. S6), although the knockdown efficiency of PsTry1 to PsTry3 were not verified. Not only the number of egg masses produced but also the number of eggs in each egg mass was not significantly different between the RNAi-treated group and the control group (Steel-Dwass test; *P* > 0.05; Fig. 5A,B). Conspicuously, egg hatching rate was reduced by maternal RNAi of *PsMCO2* but not by maternal RNAi of the other MCOs (Fig. 5C). It should be noted that, although *PsMCO3* is highly expressed in mature ovaries, no obvious reproductive phenotype was observed when it is downregulated, as is also the case for MCO3 of *N. lugens*^27^.

### Conclusion and perspective

The brown-winged green stinkbug *P. stali* possesses 7 multicopper oxidase (MCO) genes, *PsMCO1*–*PsMCO7*. By conducting RNAi knockdown and quantitative RT-PCR of MCO genes, we obtained the following main findings. First, as shown among broad insect taxa, MCO2 (laccase2) is required for cuticle pigmentation and sclerotization of *P. stali*. Second, eggshell pigmentation and sclerotization of *P. stali* do not require any of the MCOs, which is distinct from the cases of mosquitoes whose MCO2 (laccase2) is necessary for eggshell integrity. Third, nymphal RNAi of *PsMCO6* (*PsMCORP*) causes no lethal effect, which is different from the cases of the RNAi knockdown of MCORPs (orthologues of *PsMCO6*) in the red flour beetle and the brown rice planthopper. These results may suggest that MCO-mediated mechanisms underlying the cuticle pigmentation and sclerotization are conserved while those underpinning the eggshell integrity are not, which highlight the functional commonality and diversity of the multigene family across diverse insect taxa.

## Supplementary information

Supplementary information.
