# Supplementary material for: Diversity and function of multicopper oxidase genes in the stinkbug Plautia stali
Source: Sci Rep. 2020 Feb 26;10:3464. doi: 10.1038/s41598-020-60340-8 (PMC7044228; doi:10.1038/s41598-020-60340-8)
Supplement: Supplementary file 1 — Supplementary information. [file 41598_2020_60340_MOESM1_ESM.pdf]

Diversity and function of multicopper oxidase genes in the stinkbug *Plautia stali*

Yudai Nishide, Daisuke Kageyama, Masatsugu Hatakeyama, Kakeru Yokoi, Akiya Jouraku, Hiromitsu Tanaka, Ryuichi Koga, Ryo Futahashi, Takema Fukatsu

Correspondence and requests for materials should be addressed to Y. N. (nishiyu0@affrc.go.jp) and T. F. (t-fukatsu@aist.go.jp)

Supplemental Figures and Tables

Supplemental Figure 1. A neighbor-jointing tree of MCO genes.
Supplemental Figure 2. Effects of RNAi knockdown of PsMCO2 on third instar nymphs.
Supplemental Figure 3. Effects of RNAi knockdown of PsMCO2 on fifth instar nymphs.
Supplemental Figure 4. Efficacy of RNAi knockdown in 5th instar nymphs.
Supplemental Figure 5. Visualization of dsRNA taken into tissues and organs of P. stali.
Supplemental Figure 6. Efficacy of maternal RNAi on eggs.
Supplemental Table 1. Primer sequences for quantitative RT-PCR.
Supplemental Table 2. Primer sequences for RNAi.

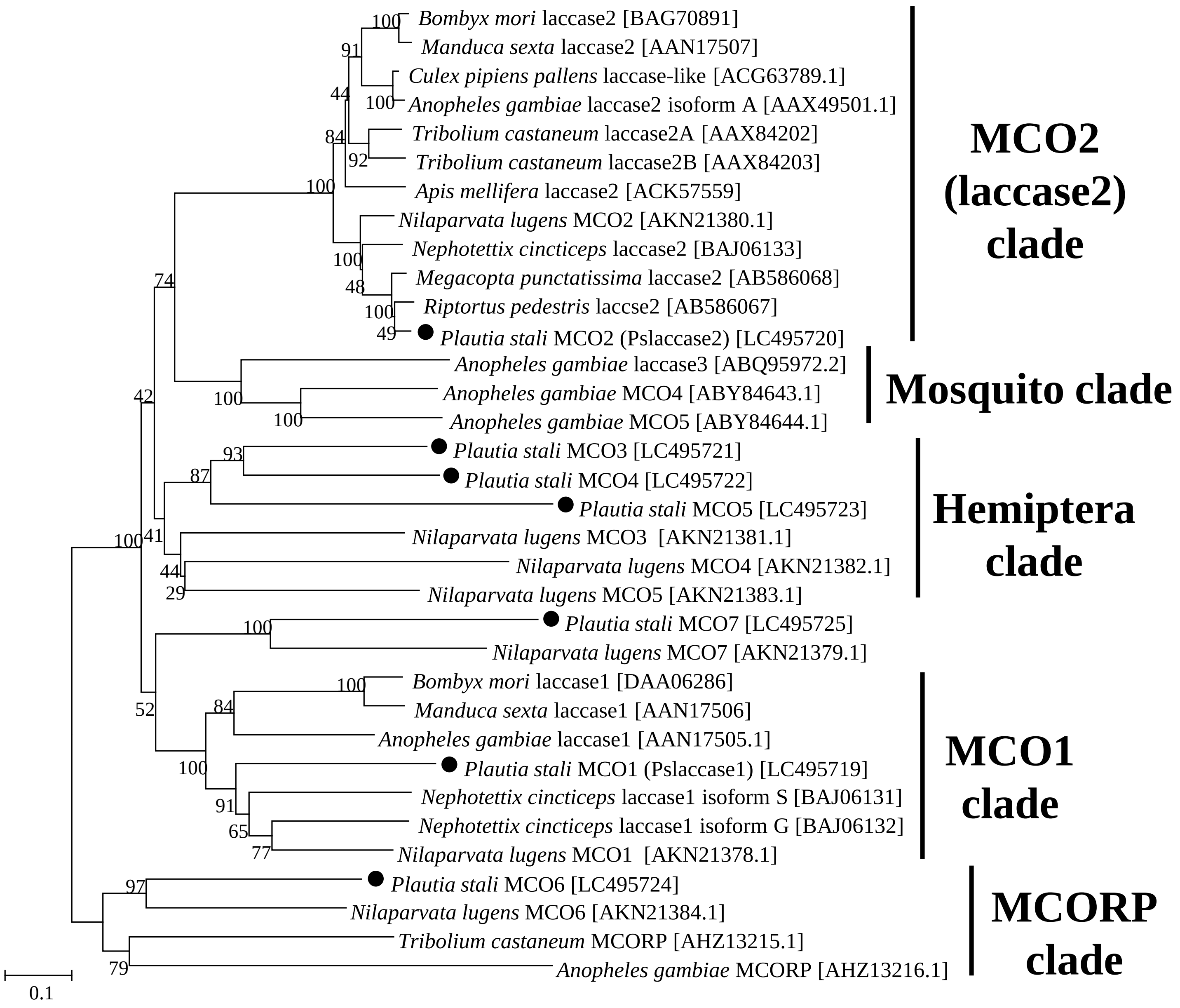

Fig. S1. A neighbor-jointing tree of MCO genes inferred from 406 aligned amino acid sites of MCO. A bootstrap probability is given on each node. The tree topology was substantially identical to the one constructed by neighbor-joining method.

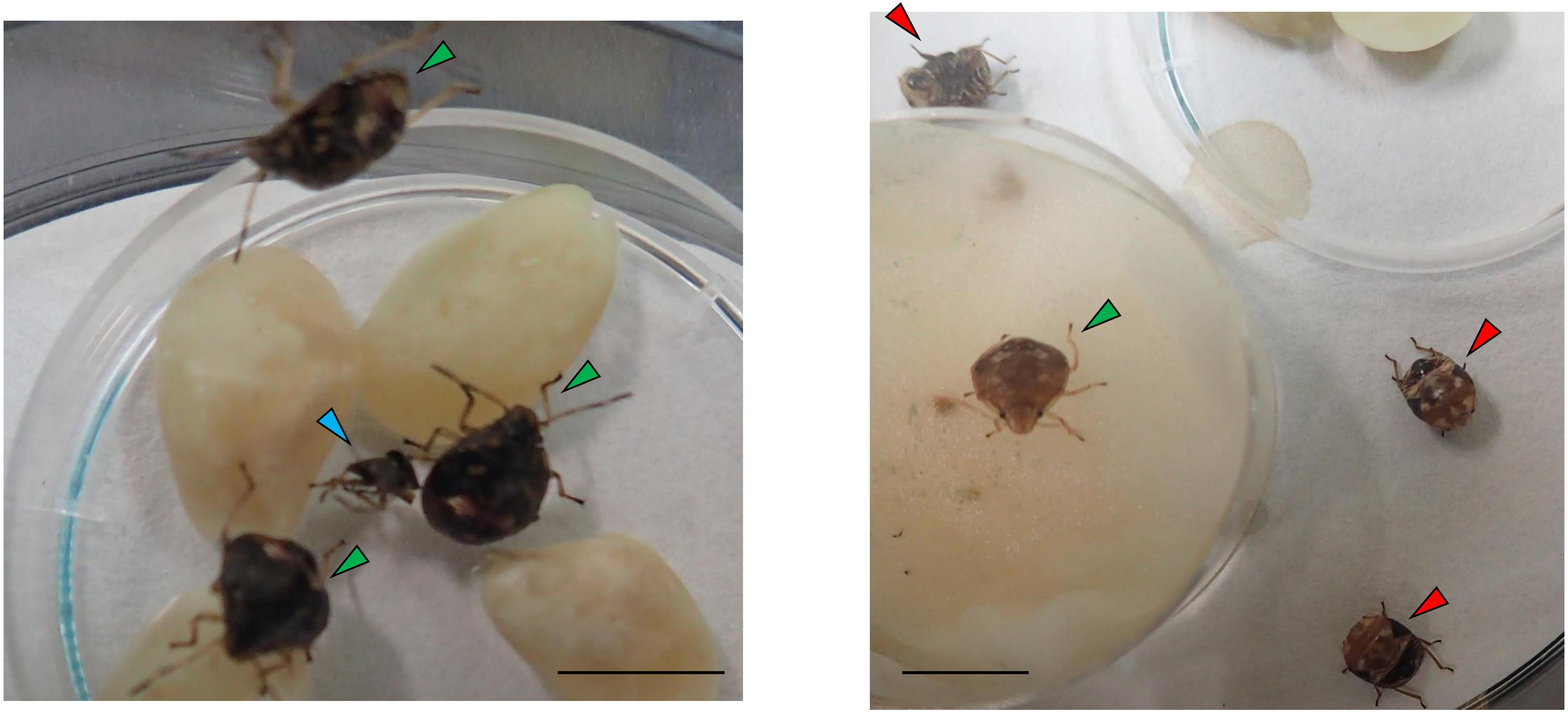

EGFP RNAi

PsMCO2 (Pslaccase2) RNAi

Fig. S2. Effects of RNAi knockdown of *PsMCO2* on third instar nymphs. Nymphs that were injected with either EGFP dsRNA as a control (left panel) or *PsMCO2* dsRNA (right panel) were shown. Arrowheads indicate 1-day-old forth instar nymphs (green), cast third instar cuticle (blue) and incomplete molting nymphs (red). Scale bars show 5 mm.

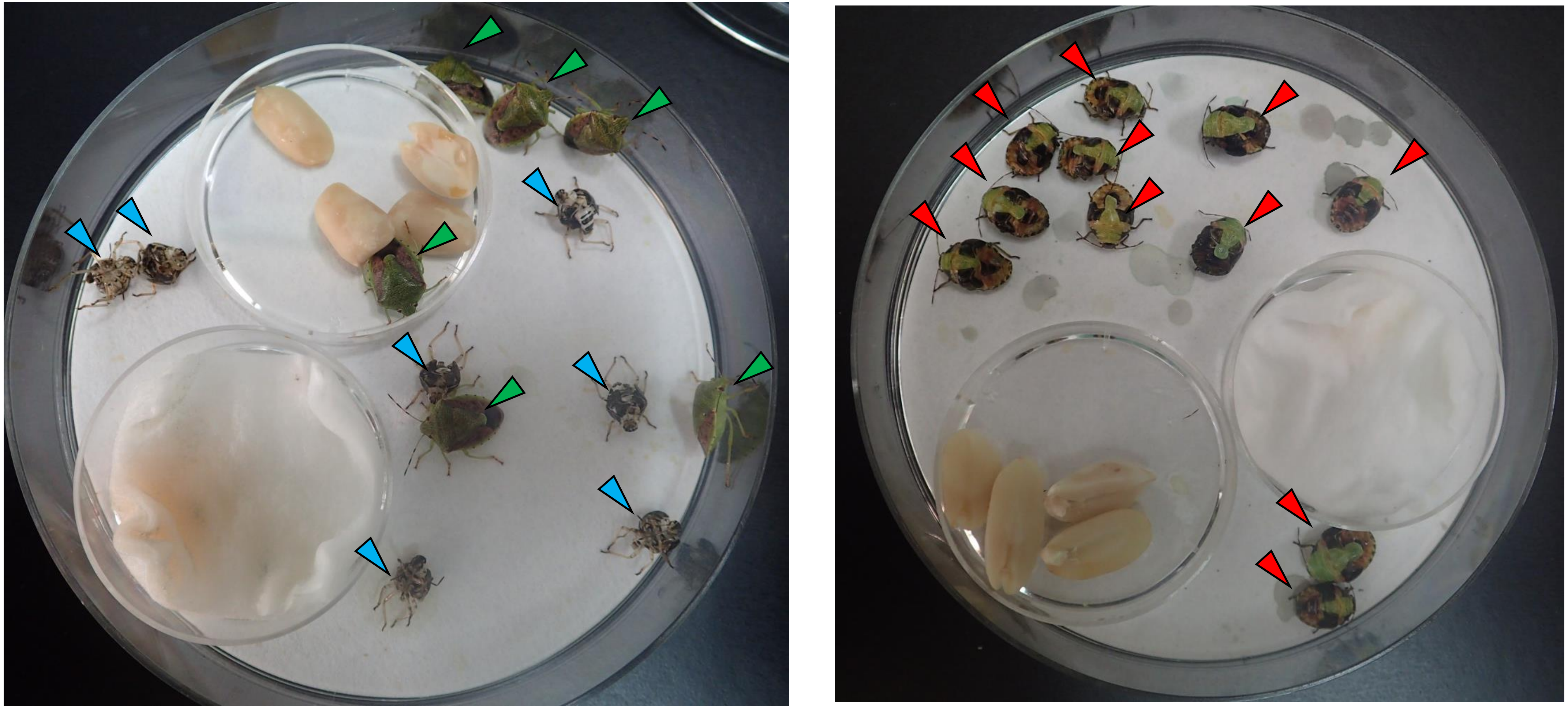

EGFP RNAi PsMCO2 (Pslaccase2) RNAi

Fig. S3. Effects of RNAi knockdown of *PsMCO2* on fifth instar nymphs. Nymphs that were injected with either EGFP dsRNA as a control (left panel) or *PsMCO2* dsRNA (right panel) were shown. Arrowheads indicate 1-day-old adults (green), cast nymphal cuticle (blue) and incomplete molting adults (red).

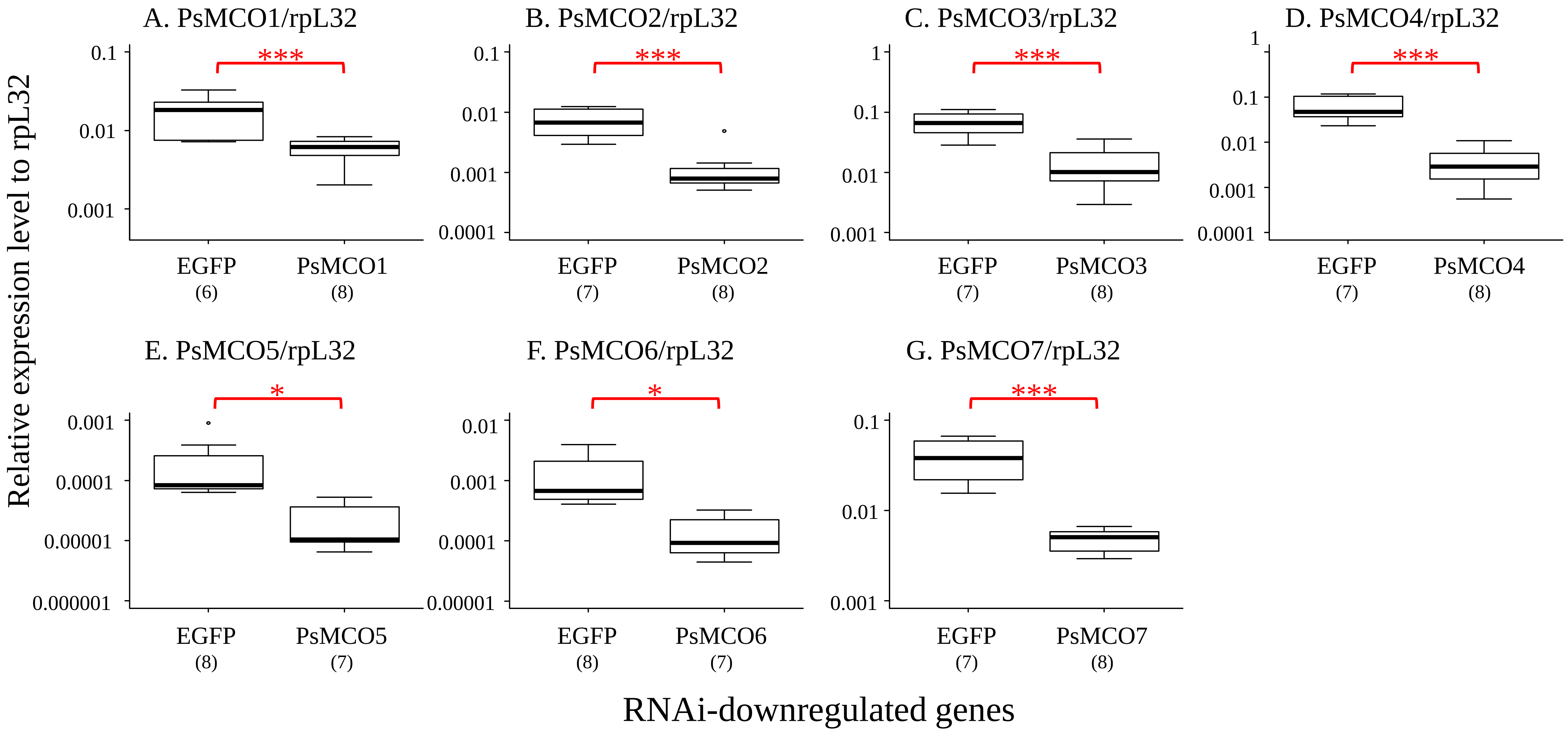

Fig. S4. Efficacy of RNAi knockdown in 5th instar nymphs. (A) *PsMCO1* (*Pslaccase1*). (B) *PsMCO2* (*Pslaccase2*). (C) *PsMCO3*. (D) *PsMCO4*. (E) *PsMCO5*. (F) *PsMCO6* (*PsMCORP*). (G) *PsMCO7*. In comparison with EGFP dsRNA injection, gene expression of each MCO decreased significantly by each MCO dsRNA injection (t-test: $P < 0.05$). Significant downregulation compared with EGFP injection is shown by asterisks (t-test: *, P < 0.05; **, P < 0.01; ***, P < 0.001). The numbers in parentheses show sample size (i.e. biological replicates).

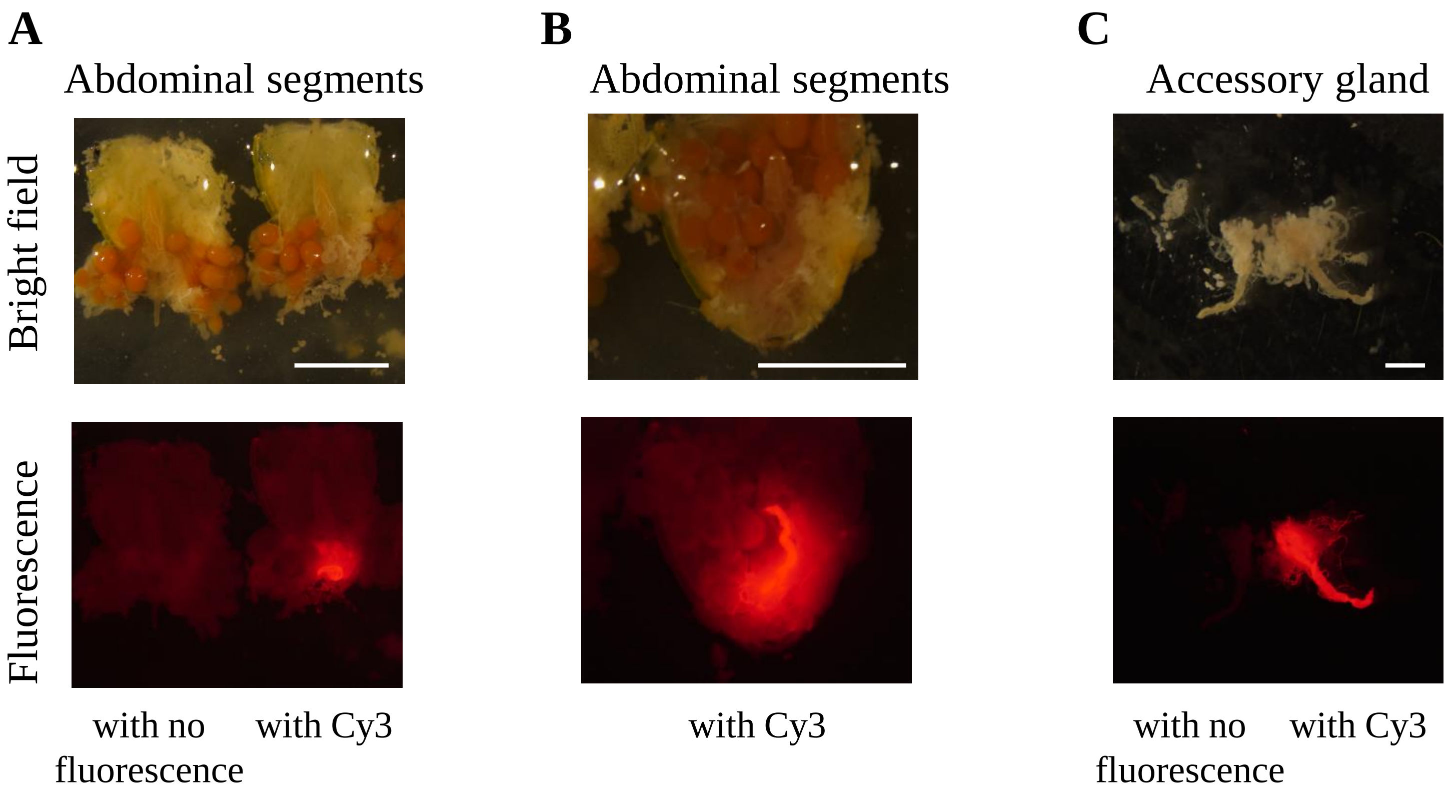

Fig. S5. Visualization of dsRNA taken into tissues and organs of *P. stali*. Non-labelled or Cy3-labelled *PsMCO2* dsRNA was injected with sexually mature females. (A) The abdominal segments, except for the gut, taken from females injected with either non-labelled dsRNA (left) or Cy3-labelled dsRNA (right). (B) By close inspection, an accessory gland-like structure was found to be intensely illuminated in the female abdomen injected with Cy3-labelled dsRNA. (C) Accessory gland-like structures were taken from females injected with either non-labelled dsRNA (left) or Cy3-labelled dsRNA (right). Upper panels show light microscopy image and lower panels show fluorescent microscopy image. Scale bars show 5 mm in (A) and (B), and 1 mm in (C).

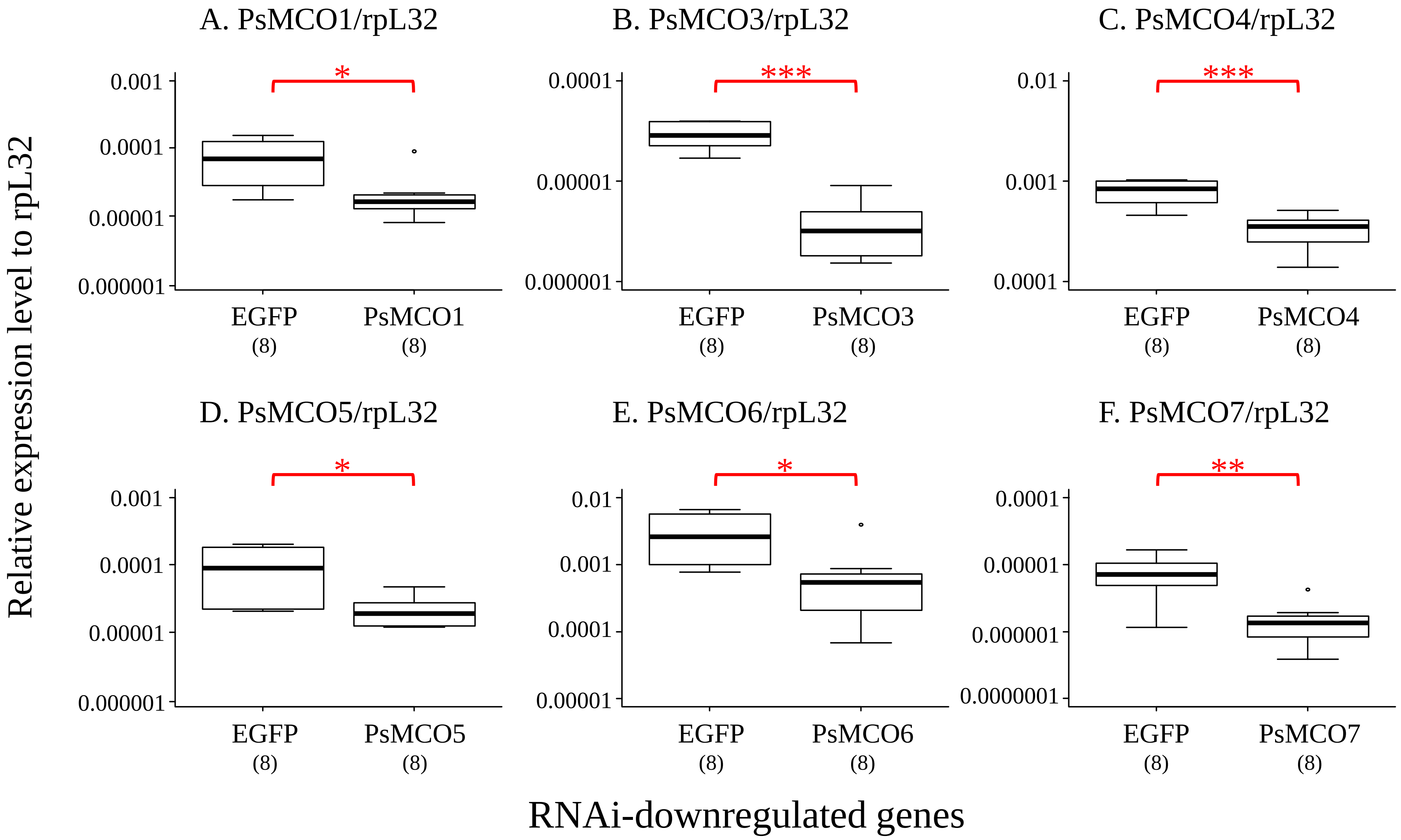

Fig. S6. Efficacy of maternal RNAi on eggs. (A) PsMCO1 (Pslaccase1). (B) PsMCO3. (C) PsMCO4. (D) PsMCO5. (E) PsMCO6 (PsMCORP). (F) PsMCO7. Egg masses laid 4 or 5 days after injection were collected and the total RNA of each egg mass was extracted 3 days after oviposition. In comparison with EGFP dsRNA injection, gene expression of each MCO decreased significantly by each MCO dsRNA injection (t-test: $P < 0.05$). Significant downregulation compared with EGFP injection is shown by asterisks (t-test: *, $P < 0.05$; **, $P < 0.01$; ***, $P < 0.001$). The numbers in parentheses show sample size (i.e. biological replicates).

Supplemental Table 1. Primer sequences for quantitative RT-PCR.

gene name	forward primer(5' to 3')	reverse primer(5' to 3')
PsMCO1 (*Pslaccase1*)	CCGGAATGTTATGTAGCGAG	TCGTATGCCACGCCTTTA
PsMCO2 (*Pslaccase2*)	CCTGCAGGGAGGGAGAA	CATTGGCAGTGACTCCAG
PsMCO3	CGCTTTCTTCGAATCTTGG	CATTGCCGATTGCTGGA
PsMCO4	GTAGCCAATACTACGA	TGAATACCTGTATGAG
PsMCO5	ACATTGGTGGTCGATGTAG	GTGGTATAGGGCACTGTG
PsMCO6 (*PsMCORP*)	TCAGGTTTGCAAGAAT	GGCACTGTGTTATCAT
PsMCO7	ATTTTTTCTGGAGAACCAAATGT	AAATACCAGGATACATAAAGCTGATA
PsrpL32	CTTCCTACTGGTTTCCGC	CGCTCTACGATTGATTTCCTTT

Supplemental Table 2. Primer sequences for RNAi.

gene name	forward primer(5' to 3')	reverse primer(5' to 3')
PsMCO1 (*Pslaccase1*)	TAATACGACTCACTATAGGGAGACAGTGTCCAGTGCTTCC	TAATACGACTCACTATAGGGAGAGGTCATCAATTGACAGCTCTAT
PsMCO2 (*Pslaccase2*)	TAATACGACTCACTATAGGGAGAACTGGCTACAGAACAAATACTC	TAATACGACTCACTATAGGGAGATCCTTTGGCATTCTTAAGTTGAT
PsMCO3	TAATACGACTCACTATAGGGAGAGCCGGCTATTCAGGTCT	TAATACGACTCACTATAGGGAGACTATCTGGAACTTGTACGATATTATCATTA
PsMCO4	TAATACGACTCACTATAGGGAGATGTCAACTCTGTCTTGATCAATTC	TAATACGACTCACTATAGGGAGAGTAGACTCGTCATAATGCTGATAAG
PsMCO5	TAATACGACTCACTATAGGGAGAATCATAACTCTTGATGGAGGAC	TAATACGACTCACTATAGGGAGAGTATGGAATATAAACCGGGTCTAAA
PsMCO6 (*PsMCORP*)	TAATACGACTCACTATAGGGAGATCCTAGTGAATGGTGTTTCTTCTAC	TAATACGACTCACTATAGGGAGACTGAGCTCTCTTATTCCTGAT
PsMCO7	TAATACGACTCACTATAGGGAGATGCCACAAATATCTCAATATCCAA	TAATACGACTCACTATAGGGAGATGAATCAATCGAAACCTGTATCTG
Pstyrosinase1	TAATACGACTCACTATAGGGAGATACCCAAGAACAGGCTTCA	TAATACGACTCACTATAGGGAGATAGGCGTGAGGAACTGAC
Pstyrosinase2	TAATACGACTCACTATAGGGAGAAGGCGTTAGAGTGACATTATTAG	TAATACGACTCACTATAGGGAGATGAGGAATTGTTCAATG
Pstyrosinase3	TAATACGACTCACTATAGGGAGAACAGTGGCGGAAACAAA	TAATACGACTCACTATAGGGAGACGGCTGCTGCTGGGTGCT
EGFP	TAATACGACTCACTATAGGGAGCTGACCCTGAAGTTCA	TAATACGACTCACTATAGGGTCCATGCCGAGAGTGATC
